# Validation of the UKPDS 82 risk equations within the Cardiff Diabetes Model

**DOI:** 10.1186/s12962-015-0038-8

**Published:** 2015-08-04

**Authors:** Philip McEwan, Thomas Ward, Hayley Bennett, Klas Bergenheim

**Affiliations:** Health Economics and Outcomes Research Ltd, Cardiff, UK; Centre for Health Economics, Swansea University, Swansea, UK; Global Health Economics and Outcomes Research, AstraZeneca, Mölndal, Sweden

**Keywords:** Cardiff Diabetes Model, Type 2 diabetes mellitus, Validation, UKPDS 82

## Abstract

**Background:**

For end-users of diabetes models that include UKPDS 82 risk equations, an important question is how well these new equations perform. Consequently, the principal objective of this study was to validate the UKPDS 82 risk equations, embedded within an established type 2 diabetes mellitus (T2DM) model, the Cardiff Diabetes Model, to contemporary T2DM outcomes studies.

**Methods:**

A total of 100 validation endpoints were simulated across treatment arms of twelve pivotal T2DM outcomes studies, simulation cohorts representing each validation study’s cohort profile were generated and intensive and conventional treatment arms were defined in the Cardiff Diabetes Model.

**Results:**

Overall the validation coefficient of determination was similar between both sets of risk equations: UKPDS 68, R^2^ = 0.851; UKPDS 82, R^2^ = 0.870. Results stratified by internal and external validation studies produced MAPE of 43.77 and 37.82%, respectively, when using UKPDS 82, and MAPE of 40.49 and 53.92%, respectively when using UKPDS 68. Areas of lack of fit, as measured by MAPE were inconsistent between sets of equations with ACCORD demonstrating a noteworthy lack of fit with UKPPDS 68 (MAPE = 170.88%) and the ADDITION study for UKPDS 82 (MAPE = 89.90%).

**Conclusions:**

This study has demonstrated that the UKPDS 82 equations exhibit similar levels of external validity to the UKPDS 68 equations with the additional benefit of enabling more diabetes related endpoints to be modeled.

## Background

The global prevalence of diabetes is estimated at 382 million and accounts for 11% ($548 billion) of the total global healthcare spend [1]. By 2035, diabetes prevalence is expected to rise by 55% to 592 million and cost the global economy $627 billion [1]. The majority of this cost is associated with the management of diabetes related macrovascular and microvascular complications: diabetes-specific therapies account for a relatively small proportion of the total cost burden, around 9–12% [2]. Nevertheless, healthcare decision makers require validated decision support tools to evaluate the expected long-term health economic benefit associated with managing diabetes. This is particularly relevant given the complexity of the diabetes specific therapeutic landscape.

Model validation in diabetes has a robust pedigree: the Mount Hood Challenge meetings are open forums for computer modelers of diabetes to discuss and compare models [3–5]. These forums allow modelers to identify important differences observed between predicted model outputs when using standardized inputs; discuss methodological challenges and agree on approaches to improving diabetes models. Diabetes models are essentially a framework designed to support a collection of risk equations and parameters; consequently, justifying the choice of equations and parameters utilised is important. A recent review identified twelve risk equations for the prediction of cardiovascular disease in subjects with type 2 diabetes mellitus (T2DM) [6]. One such set of equations are derived from the United Kingdom Prospective Diabetes Study (UKPDS), the UKPDS equations [7–9]. These equations have been used by many modeling groups for the prediction of microvascular and macrovascular risk in subjects with T2DM [10]. The recent publication of an update to the UKPDS risk equations (UKPDS 82) [11], has generated considerable interest amongst both developers and users of diabetes models. Compared to previous versions of the UKPDS equations, including the most commonly used UKPDS 68, the UKPDS 82 risk equations allow for the modeling of an expanded set of cardiovascular and microvascular endpoints; including secondary myocardial infarction and stroke events; the use of additional modifiable risk factors and mortality risk now calculated with greater granularity.

For end-users of these diabetes models that now include the UKPDS 82 update an important question is how well new equations perform. Consequently, the principal objective of this study was to validate the UKPDS 82 risk equations, embedded within an established T2DM model, to contemporary T2DM outcomes studies. A secondary objective was to illustrate the influence that the expanded risk factor set has on predicted endpoints.

## Methods

### The Cardiff Diabetes Model

The Cardiff Diabetes Model [12] is designed to estimate the long-term economic and health impact of managing patients with T2DM. The model is a fixed time increment (six-monthly) stochastic simulation with a 40-year time horizon; the core model is coded in C++ and linked to a Microsoft Excel front end. Development of the Cardiff Model began in 2003 and was initially based on the non-insulin dependent diabetes mellitus (NIDDM) model, published by Eastman [13, 14]. This model was updated to include UKPDS 56, 60 and 68 equations in 2004 and UKPDS 82 in 2014.

The model has been used to explore general health economic issues in diabetes modeling including the cost effectiveness of treatment strategies [15]; the inter-relationship haemoglobin A1c (HbA1c), hypoglycaemia and weight change on predicted quality adjusted life years [16]; the use of variance reduction techniques to improve model run-time [17]; and the influence of therapy escalation thresholds on cost-effectiveness [18]. The Cardiff model has participated in the last five Mount Hood Challenge meetings: Mount Hood 3 (2003, Oxford, UK); Mount Hood 4 (2004, Basel, Switzerland) [4]; Mount Hood 5 (2010, Malmo, Sweden) [5], Mount Hood 6 (2012, Baltimore, USA), Mount Hood 7 (2014, Stanford, USA).

In general, the model utilizes the UKPDS Outcomes Model equations (UKPDS 68 and 82) to predict macrovascular and microvascular complications in subjects with T2DM. The model is designed to evaluate a treatment and control pathway, each of which are comprised of up to three lines of therapy. Therapy escalation is either time dependent or controlled via user-defined HbA1c thresholds. The model is capable of running with mean values, with probabilistic inputs or with user-supplied patient level data; standard health economic output includes cost per life years gained and cost per quality adjusted life years (QALYs) gained. Standard model output includes time-dependent event rates, total cost and utility decrements associated with all predicted events.

### Validation studies

The validation studies included for this analysis are categorized into internal and external. Internal validation is designed to assess whether the output of the model is internally consistent with the studies and data sources used to construct the disease progression algorithm used by the model. The predominant source of data used by the Cardiff Model is the UKPDS and therefore validation to UKPDS 33 [19], 68 and 80 are considered internal validation studies. External validation compares output from the model with data not specifically used to construct the disease progression algorithms. The external validation studies selected represent a broad range including blood glucose lowering, blood pressure lowering, lipid lowering and multifactorial risk factor management. The specific external validation studies included in this analysis were: the Atorvastatin Study for Prevention of Coronary Heart Disease Endpoints in non-insulin-dependent diabetes mellitus (ASPEN) [20]; the Veterans Affairs Diabetes Trial (VADT) [21]; the Action in Diabetes and Vascular Disease: Preterax and Diamicron Modified Release Controlled Evaluation (ADVANCE) [22]; the Action to Control Cardiovascular Risk in Diabetes (ACCORD) [23]; the Anglo–Danish–Dutch Study of Intensive Treatment In People with Screen Detected Diabetes in Primary Care (ADDITION-Europe) [24]; the Anglo–Scandinavian Cardiac Outcomes Trial-Blood Pressure Lowering Arm (ASCOT) [25]; the Collaborative Atorvastatin Diabetes Study (CARDS) [26]; the Saxagliptin Assessment of Vascular Outcomes Recorded in patients with diabetes mellitus–Thrombolysis in Myocardial Infarction (SAVOR-TIMI) 53 study [27] and the look Action for Health in Diabetes (AHEAD) study [28].

### Model set-up

For each validation exercise the model’s demographics, baseline risk factor and prior event history cohort profile was initialized to each validation study’s cohort profile. Clinical events, consistently defined between the publication and those predicted by the Cardiff Model were compared over the relevant time horizon. Where appropriate, each simulated cohort had treatment effect profile applied to consistent to that reported in each respective study. These treatment profiles were fully applied in the first cycle of the model. In all subsequent cycles, risk factor trajectories were updated according to the natural history progression specified by the UKPDS 68 panel equations for HbA1c, systolic blood pressure (SBP), total cholesterol:high density lipoprotein ratio (TC:HDL). The natural history of weight gain was set to +0.1 kg per year [19] and eGFR was assumed to decline by 0.7 ml/min/year/1.73 m^2^ [29]; white blood cell count, low-density lipoprotein (LDL) cholesterol, albuminuria and heart rate were held constant using either baseline or post-treatment values (where reported).

### Risk factor uncertainty

Where baseline risk factors required by the model were not reported in the validation studies we used the Cardiff Model’s default settings, drawn from UKPDS 82 [30]. In order to better understand how the new risk factors required by UKPDS 82 risk equations impact predicted event rates we sampled baseline values from a normal distribution (±2 × standard error, as reported in UKPDS 82) which for white blood cell count was 6.9 ± 1.8 × 10^6^/ml; LDL cholesterol, 3.0 ± 0.6 mmol/l; estimated glomerular filtration rate (eGFR), 77.7 ± 15 ml min^1−^ (1.73 m)^−2^; heart rate, 72 ± 12 bpm; HbA1c, 145 ± 13 g/l. For the presence of micro or macroalbuminura (albumin ≥50 mg/l) we sampled the binomial proportion, *p*, using the respective sample size from each study and uniformly sampling the proportion with albumin ≥50 mg/l between 9 and 35%.

### Goodness of fit

There exist a number of candidate statistical tests for comparing model output with observed outcomes; however, there is no consensus upon the best approach [31]. Formal hypothesis testing is complicated by the fact that the disease model we are seeking to evaluate is only an approximation to the actual disease; consequently testing the null hypothesis of no difference between the validation study observation and model predictions makes little sense. However, while recognizing that the model is an approximation of the actual disease, we utilised linear regression analysis on annualized event rates (observed versus predicted) to formally assess model fit. Furthermore, to understand where model fit was poor, we also assessed goodness of fit between simulated and observed endpoints for trials, endpoints and treatment arm using the mean absolute percentage error (MAPE). These were calculated by comparing *X* (the predicted endpoints from the Cardiff Model) with *Y* (the observed endpoints reported in each trial): *X*_1_, *X*_2_,…, *X*_*n*_ and *Y*_1_, *Y*_2_,…, *Y*_*n*_, where *n* is the sample size (the number of validation endpoints). We define the residuals *Z* as the paired difference between the two sets of results (model and trial): *Z* = *Y* − *X*, *i* = 1, 2,…, *n*. Calculation of the MAPE was computed using:

$$MAPE = \frac{1}{n}\mathop \sum \limits_{i = 1}^{n} \left| {\left( {\frac{{Y_{i} - X_{i} }}{{Y_{i} }}} \right) \times 100} \right|$$

Finally, and consistent with other validation studies published in the health economic literature, we present scatterplots of observed versus predicted endpoints along with the coefficient of determination (*R*^*2*^).

## Results

**Fig. 1:**
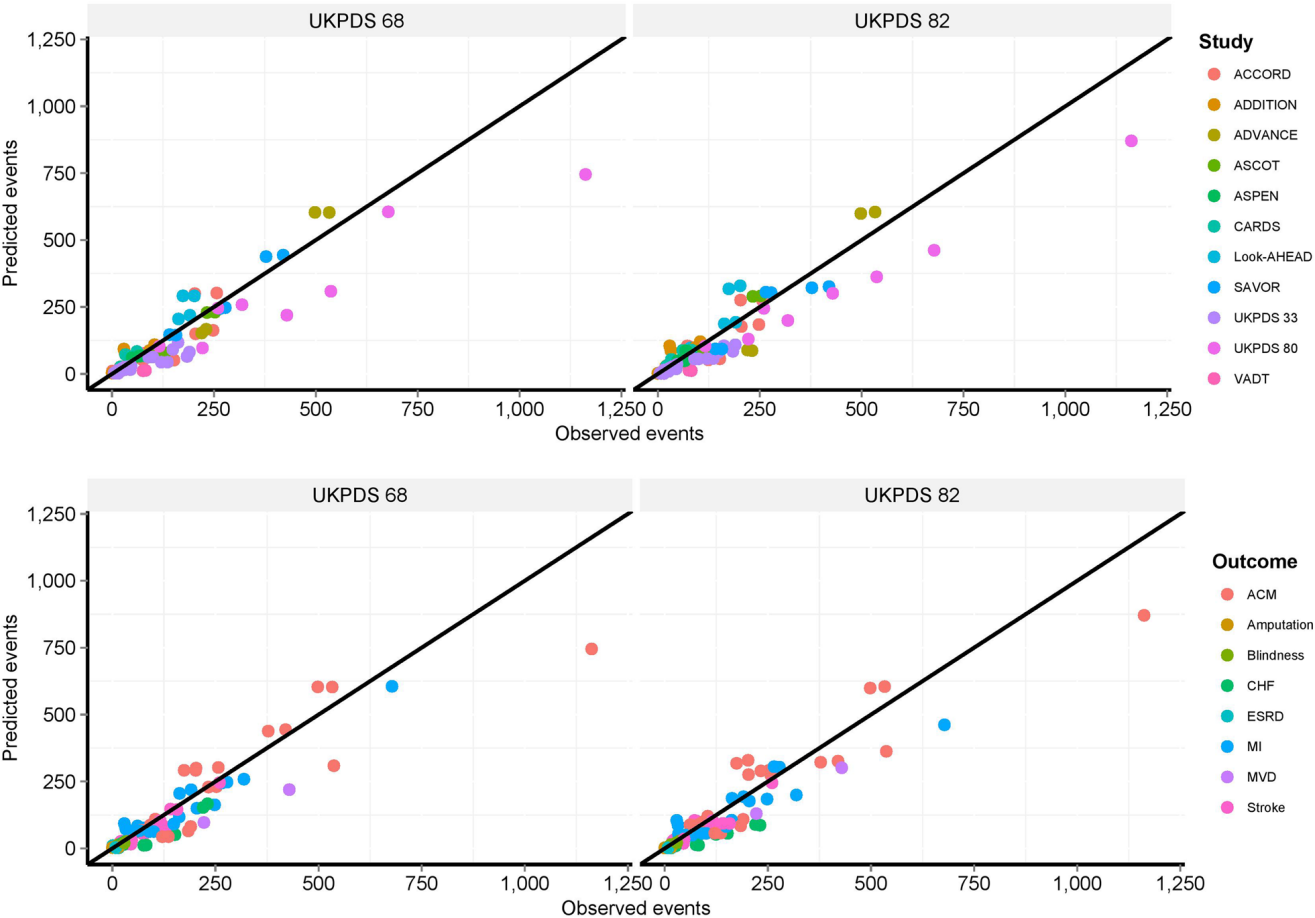
Observed versus predicted endpoints stratified by validations study, endpoint and UKPDS equations. Overall validation coefficient of determination for UKPDS 68, R^2^ = 0.851; UKPDS 82, R^2^ = 0.870. *ACM* all-cause mortality, *CHF* congestive heart failure, *CHD* coronary heart disease, *CV* cardiovascular, *MI* myocardial infarction, *ESRD* end stage renal disease, *MVD* microvascular disease, *PE* primary endpoint.

A total of 100 validation endpoints were simulated across treatment arms of twelve pivotal T2DM outcomes studies. Results from assessing overall goodness of fit via linear regression modeling to the annualized event rate is presented in Table 1. On average both sets of equations tended to slightly under predict the observed event rate as indicated by the intercept terms reported in Table 1; 3.6 (p < 0.001) for UKPDS 68 and 2.4 (p = 0.056) for UKPDS 82. The borderline non-significant intercept term and slope coefficient for UKPDS 82 indicated a slight improvement in fit compared to UKPDS 68. Across all stratifications, the *R*^*2*^ statistic showed high degrees of linear correlation between observed and predicted and points. Figure 1 illustrates the relationship between study observed versus predicted endpoints stratified by validations study, endpoint and UKPDS equations. Overall the validation coefficient of determination was similar between both sets of equations: UKPDS 68, R^2^ = 0.851; UKPDS 82, R^2^ = 0.870.

**Table 1:** Observed versus predicted events for UKPDS 68 and 82 risk equations across all studies and outcomes

	Estimate	SE	t statistic	Pr(>|t|)
UKPDS 68				
Intercept	3.603	0.959	3.757	<0.001
Expected	0.915	0.022	41.866	<0.001
UKPDS 82				
Intercept	2.422	1.251	1.936	0.056
Expected	0.999	0.031	32.166	<0.001

*Estimate* regression coefficient, *SE* standard error.

Table 2 reports MAPE and R^2^ statistics observed and predicted events overall, and by study and endpoint for both UKPDS 68 and 82 risk equations. Overall MAPE was 40.00% (UKPDS 82) and 48.99% (UKPDS 68). Results stratified by internal and external validation studies produced MAPE of 43.77 and 37.82%, respectively, when using UKPDS 82, and MAPE of 40.49 and 53.92%, respectively when using UKPDS 68. Areas of lack of fit, as measured by MAPE were inconsistent between sets of equations with ACCORD demonstrating a noteworthy lack of fit with UKPPDS 68 (MAPE = 170.88%) and the ADDITION study for UKPDS 82 (MAPE = 89.90%).

**Table 2:** Summary measure of goodness of fit for the predicted endpoints obtained from the Cardiff Diabetes Model

	UKPDS 68		UKPDS 82	
	MAPE (%)	R^2^	MAPE (%)	R^2^
Study				
ACCORD	170.88	0.69	50.06	0.73
ADDITION	69.83	0.68	89.90	0.67
ADVANCE	23.13	0.99	38.91	0.99
ASCOT	17.36	0.94	20.42	0.96
ASPEN	19.06	0.86	24.66	0.81
CARDS	39.84	0.39	32.12	0.69
Look-AHEAD	27.16	0.88	29.94	075
SAVOR	8.65	0.96	22.68	0.79
UKPDS 33	43.87	0.83	48.63	0.95
UKPDS 80	28.67	0.89	26.75	0.98
VADT	39.62	0.50	31.05	0.47
Endpoint				
MI	41.80	0.93	43.68	0.85
Stroke	28.05	0.95	29.35	0.85
CHF	38.09	0.77	50.05	0.68
ACM	27.36	0.78	30.35	0.85
Amputation	48.31	0.87	48.49	0.56
Blindness	23.40	0.92	20.31	0.96
ESRD	278.20	0.78	88.11	0.66
MVD	52.58	NA	35.59	NA

*ACM* all-cause mortality, *CHF* congestive heart failure, *CHD* coronary heart disease, *CV* cardiovascular, *MI* myocardial infarction, *ESRD* end stage renal disease, *MVD* microvascular disease.

**Fig. 2:**
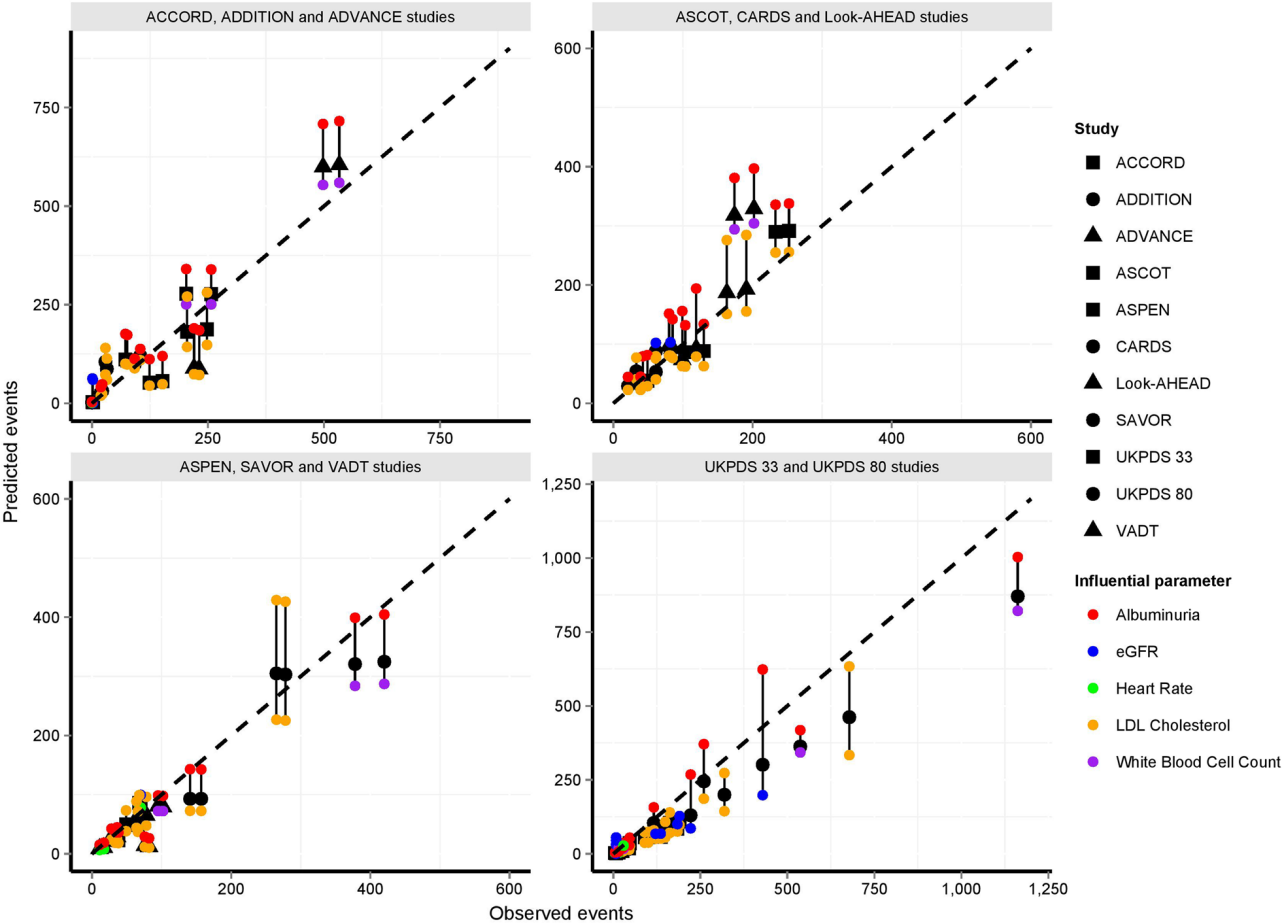
Observed versus predicted events using the UKPDS 82 risk equations. Observed (*coloured solid shapes*) versus predicted (*solid black shapes*) together with estimates of the prediction range associated with sampling baseline albuminuria, eGFR, heart rate, LDL cholesterol and white blood cell count. *Upper* and *lower* ranges are shown for each risk factor exerting most influence on prediction validation endpoint.

Figure 2 illustrates the relationship between observed versus predicted events using the UKPDS 82 risk equations together with estimates of the prediction range associated with sampling baseline albuminuria, eGFR, heart rate, LDL cholesterol and white blood cell count. Upper and lower ranges are shown for each risk factor exerting most influence on prediction validation endpoint. Of note is that plausible variation in these parameters has the potential to impact the predicted event rate considerably. In 90% of validation endpoints, sampled values within 2 standard errors of the mean (as reported by UKPDS) would have resulted in predicted endpoints lying on the 45º identity line.

## Discussion

Despite a wide range of potential risk equations available to diabetes modelers those published from UKPDS have been widely adopted by a number of modeling groups [10]. Consequently, demonstrating the predictive validity of these new equations is an important consideration for those seeking reassurance that they represent an improvement over the existing UKPDS 68 risk equations; not least because their inclusion within an existing model is not a trivial undertaking. The results from this study would suggest that the UKPDS 82 risk equations are associated with an improved fit to external validation studies compared to UKPDS 68 (MAPE of 40.0 and 49.0% for UKPDS 82 and 68, respectively) and, as expected, a modest improvement to the UKPDS study endpoints. In addition to offering an improved fit to external validation data, the UKPDS 82 equations are also capable of predicting an expanded set of diabetes related endpoints, in particular, secondary myocardial infarction, stroke and amputation events and ulcers. However, these equations require the specification of additional risk factors not always, or routinely, available; and, as illustrated in this analysis, these risk factors exert considerable influence on event rate predictions. This is of less relevance when considering the use of these equations to support cost-effectiveness analyses because it is the incremental difference in event rates (typically driven by risk factors reported in randomized clinical trials) that is important. However, even when the focus is centered on incremental results the prediction of plausible absolute event rates is still a relevant objective. Consequently, users of models employing the UKPDS 82 risk equations will need to ensure missing risk factor information is imputed with care.

In terms of validation results assessed via MAPE and the coefficient of determination (*R*^2^) the results presented in this analysis are consistent with those reported for the recent CORE diabetes model validation [32] which, for UKPDS 82, were MAPE = 39% and *R*^2^ = 0.765 compared to MAPE = 40.0% and *R*^2^ = 0.870 in this study. A recently published validation of the IHE Cohort Model reported an *R*^2^ of 0.968 for the UKPDS 82 risk equations and also reported a tendency for the equations to systematically under predict event rates [33]. Our analysis did not identify any systematic bias associated with the UKPDS 82 equations; however, any differences may reflect findings due to the choice of validation studies used and baseline values specified for albuminuria, eGFR, heart rate, LDL cholesterol and white blood cell count. Furthermore, it is important to emphasize that in conducting these validation exercises no attempt was made to calibrate the output of the model to each individual trial. The model was initialized with baseline characteristics as reported in each trial and run over the appropriate time horizon. Model results were only compared with those reported after all analyses had been complete. Consequently, the validation results presented in this study are the same as those we would have presented if were blinded to the respective study publications.

ISPOR’s model validation guidelines emphasize the value of quantitatively assessing goodness of fit [34]; however, the specific choice of quantitative measure to use is not obvious. We have reported the coefficient of determination (*R*^2^) measure to facilitate a comparison due to its widespread use in the health economic model validation literature. However, we found the MAPE a more useful measure to highlight specific areas of lack-of-fit. This raises the important issue of how one should interpret any quantitative measure of goodness of fit. For example, there is no one method of ascertaining if a model’s predictions are appropriately close to the validation study endpoints. While we performed a statistical measure of goodness of fit it should be noted that this test is highly dependent on the number of validation studies performed: as the sample size increases so will the likelihood of rejecting the null hypothesis.

## Conclusions

This study has demonstrated that the UKPDS 82 equations exhibit similar levels of external validity to the UKPDS 68 equations with the additional benefit of enabling more diabetes related endpoints to be modeled. However, an area of remaining uncertainty for model developers relates to the influence of the expanded risk factor set (white blood cell count, LDL cholesterol, albuminuria, heart rate and eGFR) upon endpoint prediction. The specification of appropriate baseline values and time-dependent trajectories (where relevant) will be an important consideration and an area in which future research should be focused.

## Abbreviations

eGFR: estimated glomerular filtration rate
HbA1c: haemoglobin A 1C
LDL: low density lipoprotein
MAPE: mean absolute percentage error
NIDDM: non-insulin dependent diabetes mellitus
QALY: quality adjusted life years
T2DM: type 2 diabetes mellitus
